# An Interesting Case of Bishop-Koop Stoma Prolapse

**Published:** 2010-12-01

**Authors:** Bilal Mirza

**Affiliations:** Department of Pediatric Surgery, The Children's Hospital and the Institute of Child Health Lahore, Pakistan

A 4-month-old male baby presented with enterostomy prolapse. Past medical history revealed two operations elsewhere during third week of life. The first operation was performed for pneumoperitoneum due to necrotizing enterocolitis (NEC) of distal jejunum. The involved portion of small intestine was resected and a primary end-to-end jejuno-ileal anastomosis performed. The patient had to be re-explored due to anastomotic disruption and then an end-to-side jejuno-ileal anastomosis with Bishop-Koop ileostomy fashioned (Fig. 1). The patient remained well for three months and passed stool per rectally and occasionally from stoma.

**Figure:**
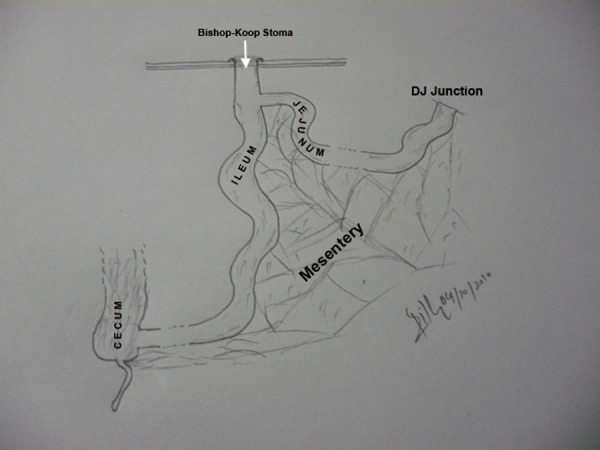
Figure 1: A line diagram illustrating end-to-side jejuno-ileal anastomosis with Bishop-Koop ileostomy.

The patient on arrival was vitally stable with normal labs. The general physical and systemic examinations were unremarkable besides a prolapsed enterostomy. Patient was anesthetized. The prolapse was inverted Y shaped, with the first limb the original Bishop Koop prolapse of ileal mucosa; whereas the second limb was the prolapsed mucosa of jejunum through end-to-side jejuno-ileal anastomosis. The mucosal anastomotic line was visible at the proximal part of that limb (Fig. 2). Initially the jejunal mucosa was returned back to the main stump followed by reduction of ileal mucosa. U-stitches were applied to hold the mucosa in place (Fig. 3). Patient was discharged after 2 days and appointment given for reversal of stoma.

**Figure:**
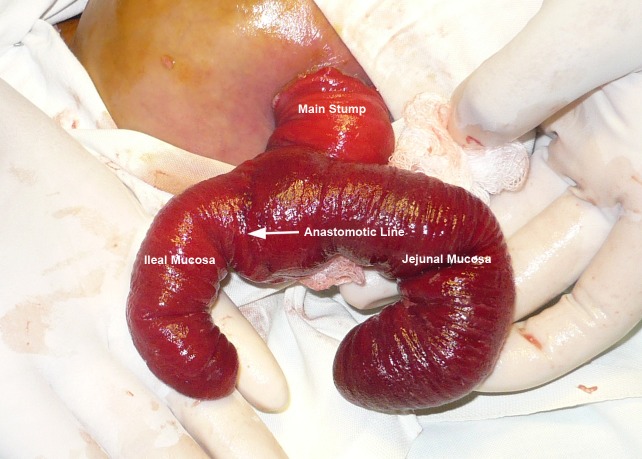
Figure 2: The Prolapse of ileal and jejunal mucosa along with anastomotic line of end-to-side jejuno-ileal anastomosis is evident.

**Figure:**
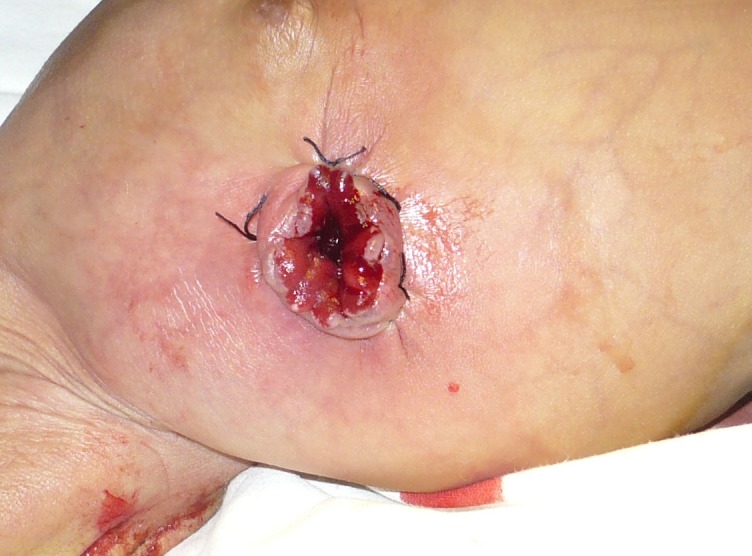
Figure 3: After reduction of prolapsed enterostomy.

## DISCUSSION

Enterostomies are commonly made for various pediatric surgical conditions. Different types of enterostomies include loop, divided/double barrel, Hartmann, santulli, Bishop-Koop etc. These may be classified as temporary or permanent depending upon the underlying condition for which they have been formed [1,2].

Bishop-Koop enterostomy was originally devised for the patients with meconium ileus, but, it has also been used for other pediatric surgical conditions such as intestinal atresia and NEC. Forming a Bishop Koop stoma involves anastomosis of end of proximal bowel to the side of distal bowel and exteriorizing the end of distal bowel as chimney -enterostomy (Fig. 1) [2,3].

The basic purpose of a Bishop-Koop enterostomy, in patients of meconium ileus, is to provide a vent for and irrigation of the distal bowel having thick inspissated meconium. In other pediatric surgical conditions, it is being used as a safety guard for intestinal anastomosis where a diversion enterostomy is not desirable like stoma in very proximal part of intestine and in conditions where intestinal length is short [3].

Enterostomies are associated with many problems such as; stoma retraction, prolapse, narrowing, peri-stomal hernia/evisceration of intestine, bleeding, skin excoriations, wound dehiscence, and so on. In one study enterostomy related complications were about 68% in children of different age groups. The incidence of prolapse in pediatric patients ranges between 3% and 25%. The incidence of stoma prolapse is higher with loop enterostomy and minimum with divided enterostomy. The highest prolapse (25%) is observed in the distal stoma of transverse loop colostomy [4].

In temporary ostomies, the stoma prolapse is usually managed conservatively, however in cases where the stoma is desired for a longer period or in case of permanent enterostomy, a revision of the stoma has been advocated [5,6].

In a perusal of English literature through “Pubmed website” using keywords “Bishop Koop” and “prolapse” no relevant paper was found. The prolapse of Bishop-Koop stoma is therefore a rare event. This may be due to a very small caliber stoma in cases with meconium ileus where it was primarily recommended; however, in our case, NEC was the primary diagnosis thus caliber of Bishop-Koop stoma was not small. This contributed to the prolapse of not only intestine but also adjacent anastomosis.

## Footnotes

**Source of Support:** Nil

**Conflict of Interest:** None declared
